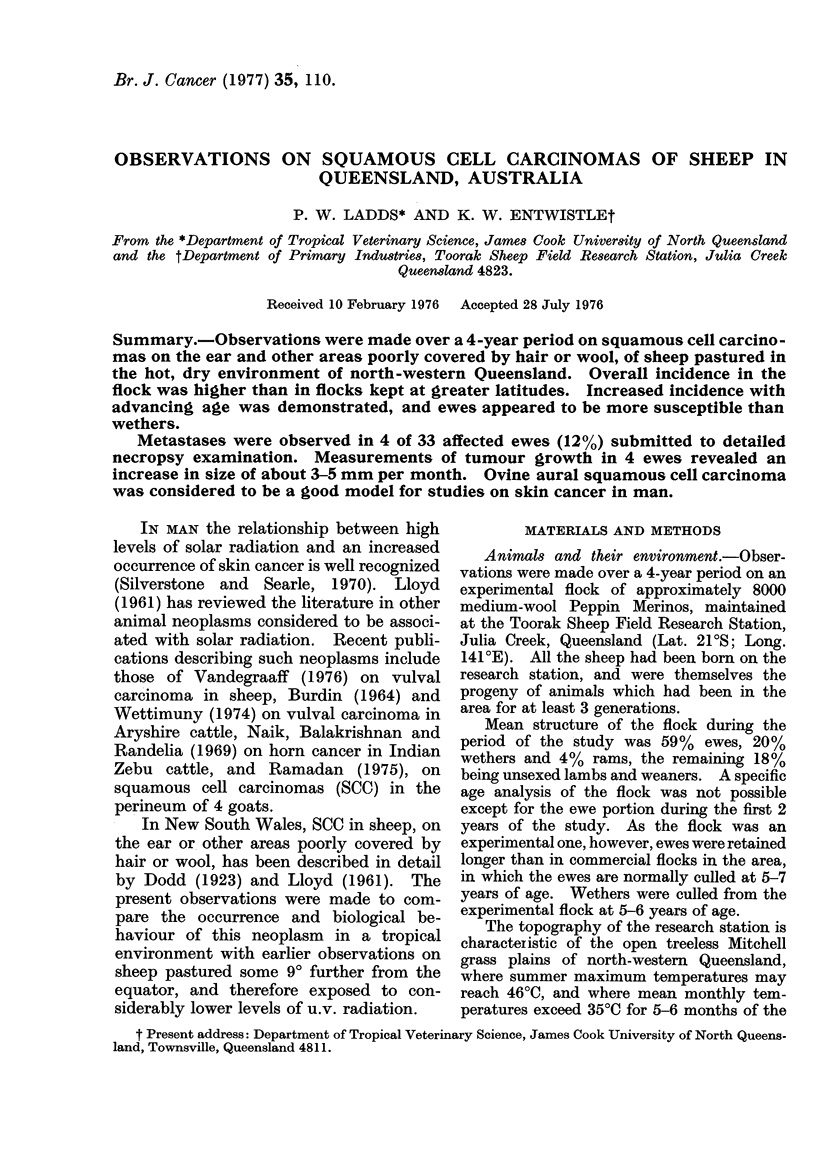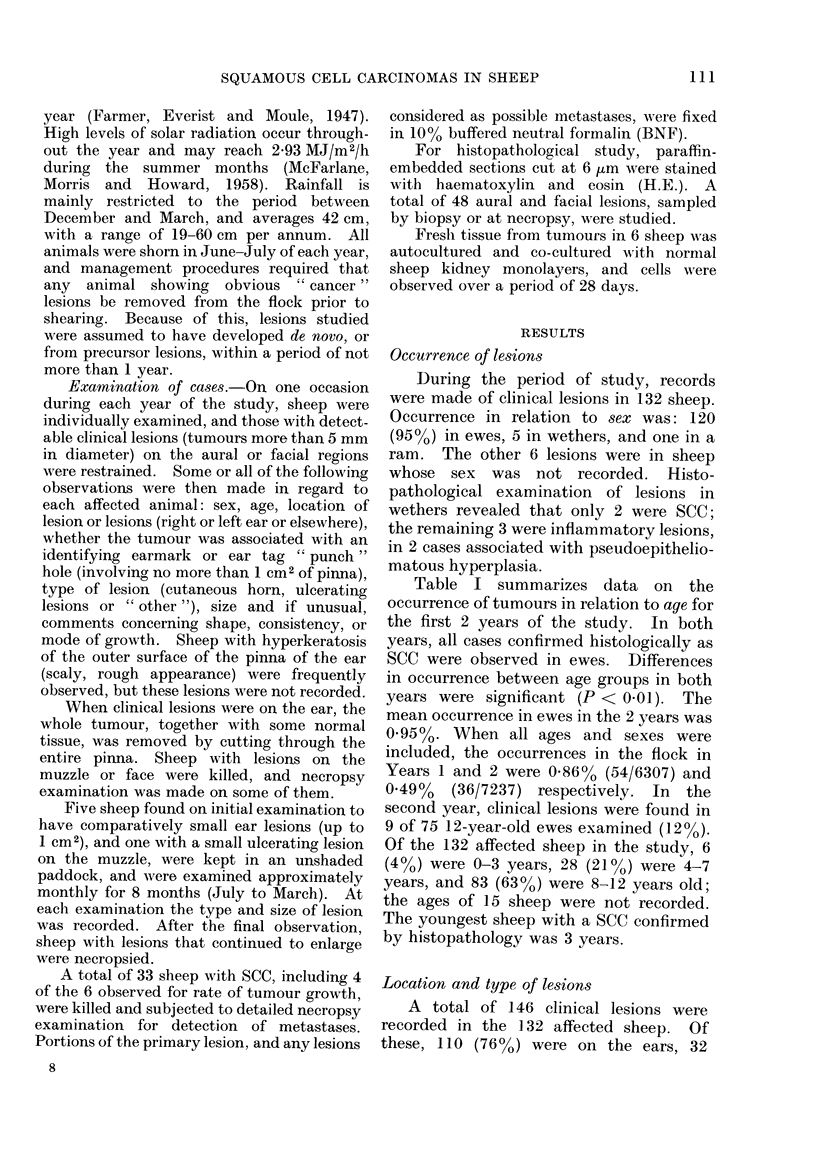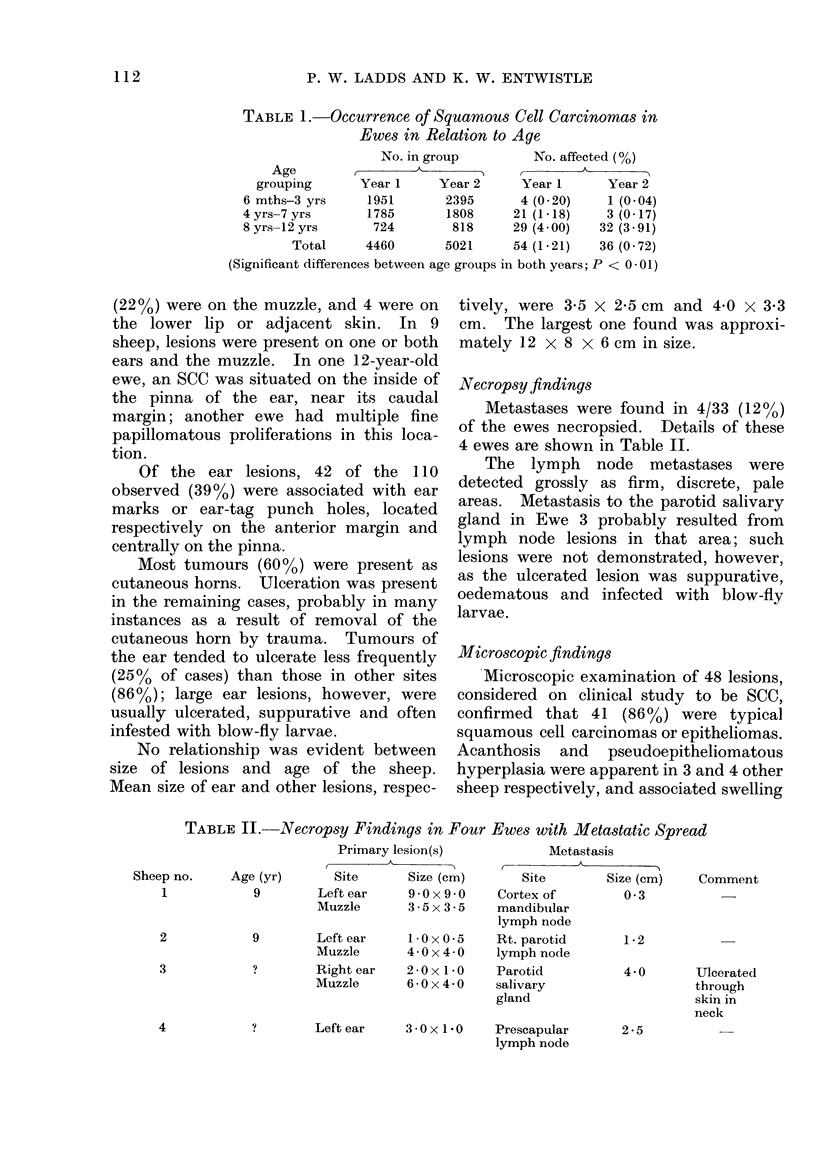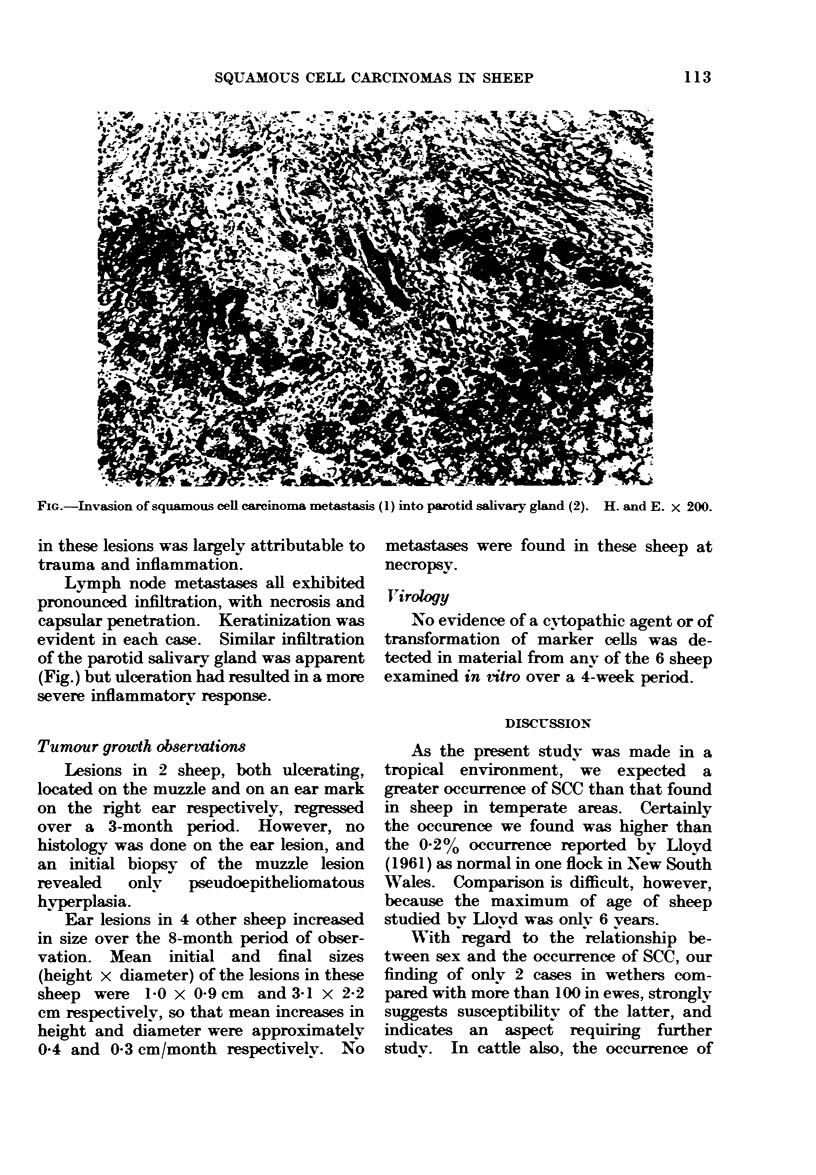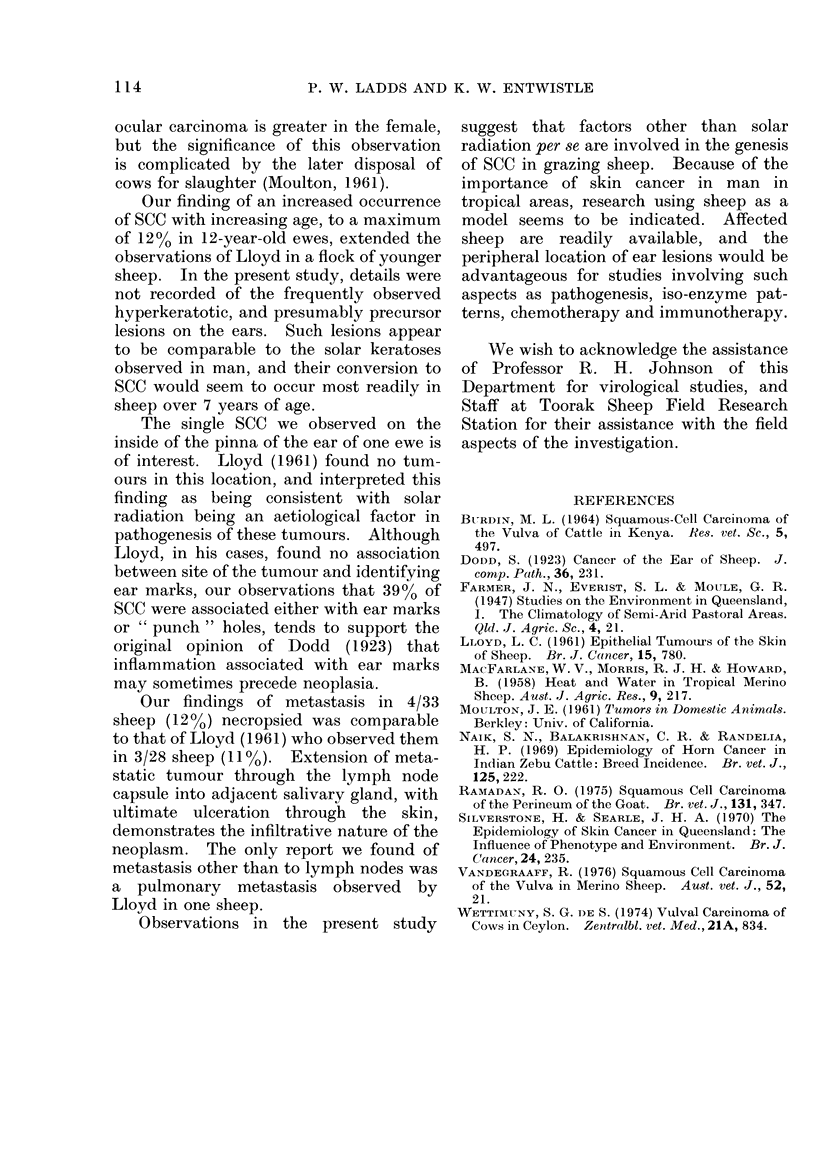# Observations on squamous cell carcinomas of sheep in Queensland, Australia.

**DOI:** 10.1038/bjc.1977.10

**Published:** 1977-01

**Authors:** P. W. Ladds, K. W. Entwistle

## Abstract

**Images:**


*Br. J. Cancer* (1977) **35**, 110.

### OBSERVATIONS ON SQUAMOUS CELL CARCINOMAS OF SHEEP IN QUEENSLAND, AUSTRALIA

P. W. LADDS* AND K. W. ENTWISTLE†

*From the *Department of Tropical Veterinary Science, James Cook University of North Queensland and the †Department of Primary Industries, Toorak Sheep Field Research Station, Julia Creek Queensland* 4823.

Received 10 February 1976    Accepted 28 July 1976

**Summary.—Observations were made over a 4-year period on squamous cell carcinomas on the ear and other areas poorly covered by hair or wool, of sheep pastured in the hot, dry environment of north-western Queensland. Overall incidence in the flock was higher than in flocks kept at greater latitudes. Increased incidence with advancing age was demonstrated, and ewes appeared to be more susceptible than wethers.**

**Metastases were observed in 4 of 33 affected ewes (12%) submitted to detailed necropsy examination. Measurements of tumour growth in 4 ewes revealed an increase in size of about 3–5 mm per month. Ovine aural squamous cell carcinoma was considered to be a good model for studies on skin cancer in man.**

In MAN the relationship between high levels of solar radiation and an increased occurrence of skin cancer is well recognized (Silverstone and Searle, 1970). Lloyd (1961) has reviewed the literature in other animal neoplasms considered to be associated with solar radiation. Recent publications describing such neoplasms include those of Vandegraaff (1976) on vulval carcinoma in sheep, Burdin (1964) and Wettimuny (1974) on vulval carcinoma in Aryshire cattle, Naik, Balakrishnan and Randelia (1969) on horn cancer in Indian Zebu cattle, and Ramadan (1975), on squamous cell carcinomas (SCC) in the perineum of 4 goats.

In New South Wales, SCC in sheep, on the ear or other areas poorly covered by hair or wool, has been described in detail by Dodd (1923) and Lloyd (1961). The present observations were made to compare the occurrence and biological behaviour of this neoplasm in a tropical environment with earlier observations on sheep pastured some 9° further from the equator, and therefore exposed to considerably lower levels of u.v. radiation.

### MATERIALS AND METHODS

*Animals and their environment.*—Observations were made over a 4-year period on an experimental flock of approximately 8000 medium-wool Peppin Merinos, maintained at the Toorak Sheep Field Research Station, Julia Creek, Queensland (Lat. 21°S; Long. 141°E). All the sheep had been born on the research station, and were themselves the progeny of animals which had been in the area for at least 3 generations.

Mean structure of the flock during the period of the study was 59% ewes, 20% wethers and 4% rams, the remaining 18% being unsexed lambs and weaners. A specific age analysis of the flock was not possible except for the ewe portion during the first 2 years of the study. As the flock was an experimental one, however, ewes were retained longer than in commercial flocks in the area, in which the ewes are normally culled at 5–7 years of age. Wethers were culled from the experimental flock at 5–6 years of age.

The topography of the research station is characteristic of the open treeless Mitchell grass plains of north-western Queensland, where summer maximum temperatures may reach 46°C, and where mean monthly temperatures exceed 35°C for 5–6 months of the

† Present address: Department of Tropical Veterinary Science, James Cook University of North Queensland, Townsville, Queensland 4811.

year (Farmer, Everist and Moule, 1947). High levels of solar radiation occur throughout the year and may reach 2·93 MJ/m²/h during the summer months (McFarlane, Morris and Howard, 1958). Rainfall is mainly restricted to the period between December and March, and averages 42 cm, with a range of 19–60 cm per annum. All animals were shorn in June–July of each year, and management procedures required that any animal showing obvious "cancer" lesions be removed from the flock prior to shearing. Because of this, lesions studied were assumed to have developed *de novo*, or from precursor lesions, within a period of not more than 1 year.

*Examination of cases*.—On one occasion during each year of the study, sheep were individually examined, and those with detectable clinical lesions (tumours more than 5 mm in diameter) on the aural or facial regions were restrained. Some or all of the following observations were then made in regard to each affected animal: sex, age, location of lesion or lesions (right or left ear or elsewhere), whether the tumour was associated with an identifying earmark or ear tag "punch" hole (involving no more than 1 cm² of pinna), type of lesion (cutaneous horn, ulcerating lesions or "other"), size and if unusual, comments concerning shape, consistency, or mode of growth. Sheep with hyperkeratosis of the outer surface of the pinna of the ear (scaly, rough appearance) were frequently observed, but these lesions were not recorded.

When clinical lesions were on the ear, the whole tumour, together with some normal tissue, was removed by cutting through the entire pinna. Sheep with lesions on the muzzle or face were killed, and necropsy examination was made on some of them.

Five sheep found on initial examination to have comparatively small ear lesions (up to 1 cm²), and one with a small ulcerating lesion on the muzzle, were kept in an unshaded paddock, and were examined approximately monthly for 8 months (July to March). At each examination the type and size of lesion was recorded. After the final observation, sheep with lesions that continued to enlarge were necropsied.

A total of 33 sheep with SCC, including 4 of the 6 observed for rate of tumour growth, were killed and subjected to detailed necropsy examination for detection of metastases. Portions of the primary lesion, and any lesions considered as possible metastases, were fixed in 10% buffered neutral formalin (BNF).

For histopathological study, paraffin-embedded sections cut at 6 $\mu$m were stained with haematoxylin and eosin (H.E.). A total of 48 aural and facial lesions, sampled by biopsy or at necropsy, were studied.

Fresh tissue from tumours in 6 sheep was autocultured and co-cultured with normal sheep kidney monolayers, and cells were observed over a period of 28 days.

### RESULTS

### *Occurrence of lesions*

During the period of study, records were made of clinical lesions in 132 sheep. Occurrence in relation to *sex* was: 120 (95%) in ewes, 5 in wethers, and one in a ram. The other 6 lesions were in sheep whose sex was not recorded. Histopathological examination of lesions in wethers revealed that only 2 were SCC; the remaining 3 were inflammatory lesions, in 2 cases associated with pseudoepitheliomatous hyperplasia.

Table I summarizes data on the occurrence of tumours in relation to *age* for the first 2 years of the study. In both years, all cases confirmed histologically as SCC were observed in ewes. Differences in occurrence between age groups in both years were significant ($P < 0{\cdot}01$). The mean occurrence in ewes in the 2 years was 0·95%. When all ages and sexes were included, the occurrences in the flock in Years 1 and 2 were 0·86% (54/6307) and 0·49% (36/7237) respectively. In the second year, clinical lesions were found in 9 of 75 12-year-old ewes examined (12%). Of the 132 affected sheep in the study, 6 (4%) were 0–3 years, 28 (21%) were 4–7 years, and 83 (63%) were 8–12 years old; the ages of 15 sheep were not recorded. The youngest sheep with a SCC confirmed by histopathology was 3 years.

### *Location and type of lesions*

A total of 146 clinical lesions were recorded in the 132 affected sheep. Of these, 110 (76%) were on the ears, 32

TABLE I.—*Occurrence of Squamous Cell Carcinomas in Ewes in Relation to Age*

| Age grouping | No. in group | | No. affected (%) | |
|---|---|---|---|---|
| | Year 1 | Year 2 | Year 1 | Year 2 |
| 6 mths–3 yrs | 1951 | 2395 | 4 (0·20) | 1 (0·04) |
| 4 yrs–7 yrs | 1785 | 1808 | 21 (1·18) | 3 (0·17) |
| 8 yrs–12 yrs | 724 | 818 | 29 (4·00) | 32 (3·91) |
| Total | 4460 | 5021 | 54 (1·21) | 36 (0·72) |

(Significant differences between age groups in both years; $P < 0{\cdot}01$)

(22%) were on the muzzle, and 4 were on the lower lip or adjacent skin. In 9 sheep, lesions were present on one or both ears and the muzzle. In one 12-year-old ewe, an SCC was situated on the inside of the pinna of the ear, near its caudal margin; another ewe had multiple fine papillomatous proliferations in this location.

Of the ear lesions, 42 of the 110 observed (39%) were associated with ear marks or ear-tag punch holes, located respectively on the anterior margin and centrally on the pinna.

Most tumours (60%) were present as cutaneous horns. Ulceration was present in the remaining cases, probably in many instances as a result of removal of the cutaneous horn by trauma. Tumours of the ear tended to ulcerate less frequently (25% of cases) than those in other sites (86%); large ear lesions, however, were usually ulcerated, suppurative and often infested with blow-fly larvae.

No relationship was evident between size of lesions and age of the sheep. Mean size of ear and other lesions, respectively, were 3·5 × 2·5 cm and 4·0 × 3·3 cm. The largest one found was approximately 12 × 8 × 6 cm in size.

### *Necropsy findings*

Metastases were found in 4/33 (12%) of the ewes necropsied. Details of these 4 ewes are shown in Table II.

The lymph node metastases were detected grossly as firm, discrete, pale areas. Metastasis to the parotid salivary gland in Ewe 3 probably resulted from lymph node lesions in that area; such lesions were not demonstrated, however, as the ulcerated lesion was suppurative, oedematous and infected with blow-fly larvae.

### *Microscopic findings*

Microscopic examination of 48 lesions, considered on clinical study to be SCC, confirmed that 41 (86%) were typical squamous cell carcinomas or epitheliomas. Acanthosis and pseudoepitheliomatous hyperplasia were apparent in 3 and 4 other sheep respectively, and associated swelling

TABLE II.—*Necropsy Findings in Four Ewes with Metastatic Spread*

| Sheep no. | Age (yr) | Primary lesion(s) | | Metastasis | | Comment |
|---|---|---|---|---|---|---|
| | | Site | Size (cm) | Site | Size (cm) | |
| 1 | 9 | Left ear<br>Muzzle | 9·0×9·0<br>3·5×3·5 | Cortex of mandibular lymph node | 0·3 | — |
| 2 | 9 | Left ear<br>Muzzle | 1·0×0·5<br>4·0×4·0 | Rt. parotid lymph node | 1·2 | — |
| 3 | ? | Right ear<br>Muzzle | 2·0×1·0<br>6·0×4·0 | Parotid salivary gland | 4·0 | Ulcerated through skin in neck |
| 4 | ? | Left ear | 3·0×1·0 | Prescapular lymph node | 2·5 | — |

FIG.—Invasion of squamous cell carcinoma metastasis (1) into parotid salivary gland (2). H. and E. × 200.

in these lesions was largely attributable to trauma and inflammation.

Lymph node metastases all exhibited pronounced infiltration, with necrosis and capsular penetration. Keratinization was evident in each case. Similar infiltration of the parotid salivary gland was apparent (Fig.) but ulceration had resulted in a more severe inflammatory response.

*Tumour growth observations*

Lesions in 2 sheep, both ulcerating, located on the muzzle and on an ear mark on the right ear respectively, regressed over a 3-month period. However, no histology was done on the ear lesion, and an initial biopsy of the muzzle lesion revealed only pseudoepitheliomatous hyperplasia.

Ear lesions in 4 other sheep increased in size over the 8-month period of observation. Mean initial and final sizes (height × diameter) of the lesions in these sheep were 1·0 × 0·9 cm and 3·1 × 2·2 cm respectively, so that mean increases in height and diameter were approximately 0·4 and 0·3 cm/month respectively. No metastases were found in these sheep at necropsy.

*Virology*

No evidence of a cytopathic agent or of transformation of marker cells was detected in material from any of the 6 sheep examined *in vitro* over a 4-week period.

DISCUSSION

As the present study was made in a tropical environment, we expected a greater occurrence of SCC than that found in sheep in temperate areas. Certainly the occurence we found was higher than the 0·2% occurrence reported by Lloyd (1961) as normal in one flock in New South Wales. Comparison is difficult, however, because the maximum of age of sheep studied by Lloyd was only 6 years.

With regard to the relationship between sex and the occurrence of SCC, our finding of only 2 cases in wethers compared with more than 100 in ewes, strongly suggests susceptibility of the latter, and indicates an aspect requiring further study. In cattle also, the occurrence of ocular carcinoma is greater in the female, but the significance of this observation is complicated by the later disposal of cows for slaughter (Moulton, 1961).

Our finding of an increased occurrence of SCC with increasing age, to a maximum of 12% in 12-year-old ewes, extended the observations of Lloyd in a flock of younger sheep. In the present study, details were not recorded of the frequently observed hyperkeratotic, and presumably precursor lesions on the ears. Such lesions appear to be comparable to the solar keratoses observed in man, and their conversion to SCC would seem to occur most readily in sheep over 7 years of age.

The single SCC we observed on the inside of the pinna of the ear of one ewe is of interest. Lloyd (1961) found no tumours in this location, and interpreted this finding as being consistent with solar radiation being an aetiological factor in pathogenesis of these tumours. Although Lloyd, in his cases, found no association between site of the tumour and identifying ear marks, our observations that 39% of SCC were associated either with ear marks or " punch " holes, tends to support the original opinion of Dodd (1923) that inflammation associated with ear marks may sometimes precede neoplasia.

Our findings of metastasis in 4/33 sheep (12%) necropsied was comparable to that of Lloyd (1961) who observed them in 3/28 sheep (11%). Extension of metastatic tumour through the lymph node capsule into adjacent salivary gland, with ultimate ulceration through the skin, demonstrates the infiltrative nature of the neoplasm. The only report we found of metastasis other than to lymph nodes was a pulmonary metastasis observed by Lloyd in one sheep.

Observations in the present study suggest that factors other than solar radiation *per se* are involved in the genesis of SCC in grazing sheep. Because of the importance of skin cancer in man in tropical areas, research using sheep as a model seems to be indicated. Affected sheep are readily available, and the peripheral location of ear lesions would be advantageous for studies involving such aspects as pathogenesis, iso-enzyme patterns, chemotherapy and immunotherapy.

We wish to acknowledge the assistance of Professor R. H. Johnson of this Department for virological studies, and Staff at Toorak Sheep Field Research Station for their assistance with the field aspects of the investigation.

### REFERENCES

Burdin, M. L. (1964) Squamous-Cell Carcinoma of the Vulva of Cattle in Kenya. *Res. vet. Sc.*, **5**, 497.

Dodd, S. (1923) Cancer of the Ear of Sheep. *J. comp. Path.*, **36**, 231.

Farmer, J. N., Everist, S. L. & Moule, G. R. (1947) Studies on the Environment in Queensland, I. The Climatology of Semi-Arid Pastoral Areas. *Qld. J. Agric. Sc.*, **4**, 21.

Lloyd, L. C. (1961) Epithelial Tumours of the Skin of Sheep. *Br. J. Cancer*, **15**, 780.

MacFarlane, W. V., Morris, R. J. H. & Howard, B. (1958) Heat and Water in Tropical Merino Sheep. *Aust. J. Agric. Res.*, **9**, 217.

Moulton, J. E. (1961) *Tumors in Domestic Animals.* Berkley: Univ. of California.

Naik, S. N., Balakrishnan, C. R. & Randelia, H. P. (1969) Epidemiology of Horn Cancer in Indian Zebu Cattle: Breed Incidence. *Br. vet. J.*, **125**, 222.

Ramadan, R. O. (1975) Squamous Cell Carcinoma of the Perineum of the Goat. *Br. vet. J.*, **131**, 347.

Silverstone, H. & Searle, J. H. A. (1970) The Epidemiology of Skin Cancer in Queensland: The Influence of Phenotype and Environment. *Br. J. Cancer*, **24**, 235.

Vandegraaff, R. (1976) Squamous Cell Carcinoma of the Vulva in Merino Sheep. *Aust. vet. J.*, **52**, 21.

Wettimuny, S. G. de S. (1974) Vulval Carcinoma of Cows in Ceylon. *Zentralbl. vet. Med.*, **21A**, 834.